# Alcohol and the workplace: the experience of Tuscan Alcohological Centre (CAR) training on early identification and brief intervention methodology

**DOI:** 10.1186/1940-0640-8-S1-A42

**Published:** 2013-09-04

**Authors:** Ilaria Londi, Tiziana Fanucchi, Gabriele Magri, Fiorella Alunni, Mariangela Spampinato, Valentino Patussi, Gianni Testino, Emanuele Scafato

**Affiliations:** 1 Tuscan Alcohological Centre-Universitary Hospital Careggi, Firenze, Italy; 2 Alcohol Regional Centre of Liguria- IRCCS Universitary Hospital San Martino-IST, Genova, Italy; 3 National Observatory on Alcohol CNESPS, WHO Collaborative Center for Research on Alcohol, National Institute of Health, Roma, Italy

## 

The Tuscan Alcohological Centre (CAR) since 2000 contributes to the prevention of alcohol-related problems. Between 2009 and 2011, the CAR has coordinated the national project "Education on the early identification and brief intervention for the prevention of problems and alcohol-related harm in the workplace and in basic health care" sponsored by the Health Ministry and entrusted to the Region of Tuscany, addressed to occupational physicians and practitioners of safety in the workplace. This paper aims to present the results of the national project, in particular, we evaluated the effectiveness of training and the satisfaction of the participants, both at the national and regional levels. The national project involved training courses in 13 partner regions. These courses were evaluated by 3 questionnaires made by the CAR, analyzed by Statistical Package for Social Science (SPSS) statistical program. The issues discussed were: motivations and expectations, knowledge and expertise on alcohol before and after training, overall satisfaction. The national project has trained 450 workers in all Italy, especially doctors in the National Health System (NHS) that deal with safety in the workplace. The results showed a significant increase in knowledge/skills. They can use in their professional practice the tools provided by the courses (AUDIT-C). The Campania region was the most expert with this method, Marche the least but the most satisfied and motivated to continue: this region, in fact, organized other courses after the first one. The courses have increased access to the best practices for the prevention of alcohol-related risks and underlining the need to establish national guidelines.

